# Levofloxacin prophylaxis for pediatric leukemia patients: monitoring of outcomes for sustained benefit and consequences

**DOI:** 10.1017/ash.2024.81

**Published:** 2024-05-22

**Authors:** Andrea L. Davis, Alexandra M. Stevens, Julienne Brackett, Lucila Marquez, Catherine E. Foster, Adriana Sarmiento Clemente, Hannah E. Sauer, Grant T. Stimes, Judith R. Campbell

**Affiliations:** 1 Department Infection Control and Prevention, Texas Children’s Hospital, Houston, TX, USA; 2 Department of Pediatrics, Section of Hematology Oncology, Baylor College of Medicine, Houston, TX, USA; 3 Texas Children’s Cancer Center, Texas Children’s Hospital, Houston, TX, USA; 4 Department of Pediatrics, Division of Infectious Diseases, Arkansas Children’s Hospital, Little Rock, AR, USA; 5 Department of Global Pediatric Medicine, St. Jude Medical Center, Memphis, TN, USA; 6 Department of Pharmacy, Texas Children’s Hospital, Houston, TX, USA; 7 Department of Pediatrics, Division of Infectious Diseases, Baylor College of Medicine, Houston, TX, USA

## Abstract

Levofloxacin prophylaxis reduces bloodstream infections in neutropenic patients with acute myeloid leukemia or relapsed acute lymphoblastic leukemia. A retrospective, longitudinal cohort study compares incidence of bacteremia, multidrug-resistant organisms (MDRO), and *Clostridioides difficile* (CDI) between time periods of levofloxacin prophylaxis implementation. Benefits were sustained without increasing MDRO or CDI.

## Introduction

Strategies to prevent bloodstream infections (BSIs) in pediatric patients with acute myeloid leukemia (AML) and relapsed acute lymphoblastic leukemia (r-ALL) is an area of heightened interest due to the high incidence of BSI and resulting morbidity and mortality in this population.^1–3^ Bacterial prophylaxis with levofloxacin has been well studied and widely adopted since the report of the Children’s Oncology Group protocol ACCL0934, showing a decrease in bacteremia in pediatric patients with AML and r-ALL.^4^ Our initial observations on levofloxacin prophylaxis show similar reductions in National Healthcare Safety Network (NHSN) central line-associated bloodstream infection (CLABSI) and gram-negative rod (GNR) BSI without an increase in multidrug-resistant organisms (MDRO) or *Clostridioides difficile* infection (CDI).^5^ Publications on levofloxacin prophylaxis emphasize that longitudinal, systematic monitoring of outcomes is critical to balance the benefits of levofloxacin prophylaxis with the importance of antimicrobial stewardship and the potential of emergence of resistant organisms or CDI. We compare incidence of BSI, MDRO, and CDI between the initial study period after implementation of levofloxacin prophylaxis and the subsequent follow-up period.

## Methods

A levofloxacin prophylaxis practice guideline was implemented in March 2019 at Texas Children’s Hospital (Houston, TX, USA), which provides levofloxacin prophylaxis to patients with AML or r-ALL during periods of chemotherapy-induced severe neutropenia. Patients who received a hematopoietic stem cell transplant (HSCT) were excluded.^5^ Systematic monitoring of adherence and outcomes was performed to confirm continued benefits and monitor for unintended consequences.

A retrospective, longitudinal cohort study was conducted for four years after implementation of the levofloxacin prophylaxis practice guideline. A cohort of patients from the early period of implementation (March 1, 2019–February 28, 2021) was compared to a cohort of patients from the subsequent two years of implementation (March 1, 2021–February 28, 2023) to ensure sustained benefits without an increase in infections due to resistant organisms or CDI. Patients with AML and r-ALL were eligible for the practice guideline and included in this study. Electronic health records were reviewed for underlying cancer diagnosis, HSCT status, and adherence to the levofloxacin practice guideline. Primary outcomes were NHSN CLABSI and any BSI event, including present on admission and secondary bacteremia. If patients had multiple BSIs, all events were included. Any BSI event was further categorized by etiology, gram-negative rod bacteria (GNR) versus viridans group streptococci or oral flora (VGS). Two positive cultures were required to be classified as a VGS BSI event. Secondary outcomes included culture from a sterile site that was positive for MDRO, bacteremia due to a levofloxacin non-susceptible GNR organism, diarrhea associated with stool positive for *C. difficile* toxin, death, and death due to bacterial infection. CDI was determined by *C. difficile* toxin PCR only if patients had new-onset diarrhea and greater than three unformed stools in a 24-hour period, without an alternative diagnosis or laxatives. Surveillance cultures for colonization with MDRO were not included. The patient clinical course was reviewed until completion of intensive chemotherapy, HSCT, death, transfer of care, or end of the study period. All patients eligible for levofloxacin prophylaxis were included in the analysis to capture the “real-world” impact and outcomes.^5^

Primary outcomes were analyzed by risk ratio (RR) of patients with NHSN CLABSI, any BSI, and any BSI with GNR or VGS, respectively. Secondary outcomes analyzed were RR of patients with BSI due to MDRO, BSI due to levofloxacin non-susceptible GNR organism, CDI, death, and death due to a bacterial infection. The Mann-Whitney U test was used to compare the days of severe neutropenia, levofloxacin prophylaxis, and cefepime therapy. Descriptive statistics were performed on demographics and underlying diagnosis using Fisher’s exact test. STATA 18 software (College Station, Texas) was used for statistical analyses.

## Results

One hundred and fifty-four patients met inclusion criteria. Seventy-two patients in the early implementation time period were compared to eighty-two patients in the subsequent time period. Demographics were similar between both groups, except age (*p* = 0.04) and underlying cancer diagnosis was similarly distributed in both cohorts (*p* = 0.51) (Table 1). In the early time period, mean days of levofloxacin prophylaxis was 60.35 [0–180] days which was similar to the subsequent time period of 53.51 [0–191] days (*Z* = 1.22) (Table 1). Days of cefepime therapy decreased from a mean of 40.51 [0–191] days in the early time period compared to a mean of 30.73 [0–127] days in the subsequent time period (*Z* = 1.95) (Table 1). Indications for cefepime, i.e., febrile neutropenia events, were not available for analysis.

**Table 1.** Demographics and clinical characteristics

Characteristics	Number (%) of patients		
	Early implementation time period: years 1–2 after prophylaxis initiated (72 patients)	Subsequent time period: years 3–4 after prophylaxis initiated (82 patients)	*P* value
Age, median (range), years	12.2 (0.6–20.6)	12.1 (0.1–23.4)	0.04
Gender			
Male	37 (51.4)	51 (62.2)	0.20
Female	35 (48.6)	31 (37.8)	
Race			
White	60 (83.3)	66 (80.5)	0.97
African American	6 (8.3)	9 (11.0)	
Asian	5 (6.9)	6 (7.3)	
Other or Unknown	1 (1.4)	1 (1.2)	
Ethnicity			
Non-Hispanic	37 (51.4)	43 (52.4)	0.75
Hispanic	35 (48.6)	39 (47.6)	
Underlying diagnosis			
Acute myeloid leukemia	49 (59.8)	47 (65.3)	0.51
Relapsed acute lymphoblastic leukemia	33 (40.2)	25 (34.7)	
Days of severe neutropenia (white blood cell <200/µL): mean, median (range)	57.67, 47.00 (1–230)	46.61, 37.50 (0–183)	*Z* = 1.23
Total days of levofloxacin: mean, median (range)	60.35, 55.00 (0–180)	53.51, 49.00 (0–191)	*Z* = 1.22
Total days of cefepime: mean, median (range)	40.51, 34.00 (0–191)	30.73, 23.50 (0–127)	*Z* = 1.9

**Table 2.** Comparison of infection and outcomes incidence per patient in early implementation and subsequent phase cohorts of the levofloxacin prophylaxis guideline

	Early implementation time period: years 1–2 After prophylaxis initiated *n* = 72 patients *n* (%)	Subsequent time period: years 3–4 after prophylaxis initiated *n* = 82 patients *n* (%)	Risk difference (95% CI)	Risk ratio (95% CI)	*P* value
Primary outcomes					
Patients with NHSN CLABSI	27 (37.5)	22 (26.8)	−0.11 (−0.25, 0.04)	0.72 (0.45, 1.14)	0.17
Any BSI event, GNR	11 (15.3)	6 (7.3)	−0.08 (−0.18, 0.02)	0.48 (0.19, 1.23)	0.13
Any BSI event, VGS	20 (27.8)	18 (22.0)	−0.06 (−0.20, 0.08)	0.80 (0.45, 1.37)	0.46
Secondary outcomes					
MDRO	6 (8.3)	3 (3.7)	−0.05 (−0.12, 0.03)	0.44 (0.11, 1.69)	0.31
Levofloxacin non-susceptible GNR BSI events*	10 (90.9)	1 (16.7)	−0.74 (−1.09, −0.40)	0.18 (0.03, 1.11)	0.01
*C difficile*-associated diarrhea	14 (19.4)	16 (19.5)	0.00 (−0.13, .13)	1.00 (0.53, 1.91)	1.00
Died	18 (25.0)	12 (14.6)	−0.10 (−0.23, 0.02)	0.59 (0.30, 1.13)	0.15
Death due to bacterial infection	1 (5.6)	1 (8.3)	0.03 (−0.16, 0.22)	1.5 (0.10, 21.74)	1.00

Legend: *P* < 0.05 statistically significant.

BSI, bloodstream infection; GNR, gram-negative rod; VGS, viridans group streptococci or oral flora; MDRO, multidrug-resistant organism; CLABSI, central line-associated bloodstream infection.

* Percentages calculated from GNR BSI events in each time period; there were 11 GNR BSI events in the early implementation time period and 6 GNR BSI events in the subsequent time period.

We observed a similar reduction of NHSN CLABSI and any GNR BSI event as in our initial two years of monitoring (NHSN CLABSI RR 0.72 [0.45, 1.14], *p* = 0.17, any GNR BSI event RR 0.48 [0.19, 1.23], *p* = 0.13) (Table 2). There was only one case of levofloxacin non-susceptible GNR BSI in the subsequent time period compared to 10 cases in the early time period, (RR 0.18, [0.03, 1.11], *p* = 0.01) (Table 2). The proportion of levofloxacin non-susceptible GNR BSI in our cohort was reduced while the hospital-wide levofloxacin resistance was stable (Supplemental Table). The RR of any VGS BSI (RR 0.80 [0.45, 1.37], p = 0.46), MDRO (RR 0.44 [0.11, 1.69], *p* = 0.31), and CDI (RR 1.00 [0.53, 1.91], *p* = 1.00) was not impacted by levofloxacin prophylaxis (Table 2).

## Discussion

Initial publications on levofloxacin prophylaxis in pediatric patients with AML and r-ALL show a reduction of BSI in this population.^3,4^ However, the long-term impact of levofloxacin prophylaxis in pediatric patients is limited.^3–5^ In our large pediatric oncology patient population, we implemented levofloxacin prophylaxis to reduce BSI in AML and r-ALL patients and monitored the impact for four years, which is longer than other reports.^3–5^ We observed a sustained reduction in NHSN CLABSI and any GNR BSI events during four years of monitoring. The risk of CDI in this population is increased due to multiple clinical factors,^6,7^ however, during four years of monitoring, new-onset CDI remained stable. Furthermore, infections due to MDRO in our AML and r-ALL patients were also stable.

Broad-spectrum antibiotic prophylaxis remains a risk for emerging resistance, thus continued monitoring for sustained impact without increased antibiotic resistance is important to antimicrobial stewardship and infection prevention programs. We monitored for levofloxacin non-susceptible GNR BSIs, but fewer events occurred in the subsequent two years of monitoring. This varies from what is reported in predominantly adult centers.^8^ Although we report a sustained benefit, this study has several limitations. Observations are from a single pediatric center and reflect post-implementation outcomes in patients eligible for levofloxacin prophylaxis. Also, levofloxacin use within our pediatric cancer center is restricted to patients eligible for the practice guideline, which may differ from other institutions. Lastly, monitoring for resistance was from clinical cultures only and not from surveillance stool cultures; thus, we did not assess for colonization.

Although levofloxacin non-susceptible GNR BSI was rare, there remains a risk for emergence of resistance to fluoroquinolones.^8,9^ Judicious use of levofloxacin prophylaxis with evidence-based criteria in select populations is beneficial. These benefits must be balanced with continued monitoring for levofloxacin resistance, MDRO, and CDI.

## Supporting information

Davis et al. supplementary material Davis et al. supplementary material

## Supplementary material

For supplementary material accompanying this paper visit http://doi.org/10.1017/ash.2024.81. click here to view supplementary material
