# Supplementary material for: Levofloxacin prophylaxis for pediatric leukemia patients: monitoring of outcomes for sustained benefit and consequences
Source: Antimicrob Steward Healthc Epidemiol. 2024 May 22;4(1):e90. doi: 10.1017/ash.2024.81 (PMC11131004; doi:10.1017/ash.2024.81)
Supplement: Davis et al. supplementary material [file S2732494X24000810sup001.docx]

**Supplemental Table: Annual number (n) and percent (%) Susceptible to Levofloxacin, *Enterobacter species* and *E. coli* isolates at Texas Children’s Hospital 2020 - 2022**

| **Organism** | **2020**  **n (%)** | **2021**  **n (%)** | **2022**  **n (%)** |
| --- | --- | --- | --- |
| *Enterobacter aerogenes* | 43 (95) | 37 (92) | 50 (98) |
| *Enterobacter cloacae* | 142 (83) | 155 (85) | 148 (93) |
| *E. coli* | 1737 (68) | 2034 (68) | 2105 (68) |
